# Progress in Surgery

**Published:** 1902-10-04

**Authors:** 


### PROGRESS IN SURGERY.

### GENITO-URINARY.

**Tubercular Kidney.**—C. N. Dowd, New York,[1] reports an interesting example of this disease in a girl 9 years old. She was first in hospital for a cold abscess in the region of the right kidney, which healed after incision and drainage. Nine months later she was again admitted for pain in the bladder region. There was pus in the urine. She improved under urotropine. Sixteen months later she was for the third time admitted to the hospital, having had severe pain in the lower right side. Tilden Brown examined the urine obtained from each ureter by ureteral catheterisation. That from the right side contained pus and tubercle bacilli, and had a low specific gravity and percentage of urea. That from the left showed a faint trace of albumen. A few months later the right kidney was removed. Recovery was uninterrupted, and she became well and strong. At the lower pole of the kidney there was found a calcareous nodule, an inch in diameter, enclosed in a fibrous capsule. It was considered to be the remains of the tubercular inflammation which had occurred three years previously. About the middle of the cortex there was a caseous tubercular nodule, and scattered throughout the cortex were numerous miliary tubercles. The short portion of the ureter removed was healthy. Dowd remarked on the case that he found it difficult to believe that the left kidney was healthy with the right one so extensively diseased, but there was no evidence of left-sided disease. The child possessed considerable power of resisting tubercular disease, and there was good reason to hope that any tubercles in the left kidney might be cured. E. Elliot jun. said it was the rule rather than the exception in tubercular kidney for advanced disease to exist in one kidney when there were apparently no other tubercular lesions in the body. Six years previously he had removed a large tubercular kidney with several pus sacs in it. Four years later the patient gave birth to a child without disturbance, and was still apparently perfectly well. Marked improvement had followed nephrectomy in such cases even when the disease had spread to the bladder. He thought that involvement of both kidneys did not necessarily contra-indicate the removal of one. Tilden Brown mentioned a case pointing the same way.

**Partial Nephrectomy.**—B. G. A. Moynihan[2] records three cases in which partial nephrectomy was successfully performed. He alludes to the experiments of Paoli which show that if one half of the kidney be excised, and after an interval the whole of the opposite kidney be removed, the remaining half kidney is adequate to the needs of the animal. Also a case of H. Morris's is cited in which that surgeon removed one-third of one kidney from a woman who had had the other kidney previously removed in Canada for tuberculous disease. Five years subsequently this patient was in good health. Moynihan's first case was one of a solitary cyst in the lower pole of the right kidney of a woman of 42. It presented as a spherical, smooth, tense, fluctuating and extremely movable tumour. The kidney could be felt in the loin, but the attachment of the tumour to that organ was doubtful before exploration. The cyst and a portion of the kidney were removed through Langenbuch's incision. The second case was that of a man of 36 with a history of constant vomiting, pain after food, and loss of weight. A smooth round movable swelling was felt above and a little to the right of the umbilicus. A diagnosis of pyloric tumour was made. After opening the abdomen the tumour was found to be a cyst which on each side merged into a flat band continuous with the lower poles of the kidneys, *i.e.*, it was a case of horse-shoe kidney with a cyst in the connecting band. The cyst and a wedge of the kidney substance on each side were excised and the wounds in the kidney closed. Thus two separate kidneys were left, each with a sutured wound at the lower pole. The symptoms of pyloric obstruction were clearly due to the pressure of the tumour upon the duodenum. In the third case a portion of a kidney containing a myxo-sarcoma was removed under the impression that the disease was tubercular. Solitary cysts of the kidney were infrequent. A. von Brackel was able to collect the notes of only 22 cases recorded between 1865 and 1899.

[1] Annals of Surgery, March 1902, p. 413. [2] Brit. Med. Jour., Feb. 1, 1902, p. 263.

(*To be continued.*)

